# A cost-effectiveness analysis of a 10-valent pneumococcal conjugate vaccine in children in six Latin American countries

**DOI:** 10.1186/1478-7547-11-21

**Published:** 2013-08-30

**Authors:** Sebastián García Martí, Lisandro Colantonio, Ariel Bardach, Julieta Galante, Analía Lopez, Joaquín Caporale, Gerhart Knerer, Jorge Alberto Gomez, Federico Augustovski, Andrés Pichon-Riviere

**Affiliations:** 1 IECS, Instituto de Efectividad Clínica y Sanitaria, Dr. Emilio Ravignani 2024, Buenos Aires C1414CPV, Argentina; 2 Health Economics Department, GSK Biologicals, Wavre, Belgium; 3 Health Outcomes Department of Latin America, GSK Biologicals, Buenos Aires, Argentina

## Abstract

**Background:**

A recently developed 10-valent pneumococcal non-typeable *H influenzae* protein D-conjugate vaccine (PHiD-CV) is expected to afford protection against more than two thirds of isolates causing IPD in children in Latin America, and also against acute otitis media caused by both Spn and NTHi. The objective of this study is to assess the cost-effectiveness of PHiD-CV in comparison to non-vaccination in children under 10 years of age in Argentina, Brazil, Chile, Colombia, Mexico and Peru.

**Methods:**

We used a static, deterministic, compartmental simulation model. The dosing regimen considered included three vaccine doses (at 2 months, 4 months and 6 months) and a booster dose (at 13 months) (3 + 1 schedule). Model outcomes included number of cases prevented, deaths averted, quality-adjusted life-years (QALYs) gained and costs. Discount for costs and benefits of long term sequelae was done at 3.5%, and currency reported in 2008-2009 U$S varying between countries.

**Results:**

The largest effect in case prevention was observed in pneumococcal meningitis (from 27% in Peru to 47% in Colombia), neurologic sequelae after meningitis (from 38% in Peru to 65% in Brazil) and bacteremia (from 42% in Argentina to 49% in Colombia). The proportion of predicted deaths averted annually ranged from 18% in Peru to 33% in Brazil. Overall, the health benefits achieved with PHiD-CV vaccination resulted in a lower QALY loss (from 15% lower in Peru to 26% in Brazil). At a cost of USD 20 per vaccine dose, vaccination was cost-effective in all countries, from being cost saving in Chile to a maximum Incremental Cost-effectiveness Ratio of 7,088 US$ Dollars per QALY gained. Results were robust in the sensitivity analysis, and scenarios with indirect costs affected results more than those with herd immunity.

**Conclusions:**

The incorporation of the 10-valent pneumococcal conjugate vaccine into routine infant immunization programs in Latin American countries could be a cost-effective strategy to improve infant population health in the region.

## Background

Acute respiratory infections are the leading cause of death in children under 5 years of age worldwide, and around 2 million infants and young children die annually due to pneumonia [1]. The most common causative agents of bacterial pneumonia in children are *Streptococcus pneumoniae* (Spn) and *Haemophilus influenzae*; both bacteria are also responsible for many cases of sepsis, meningitis and acute otitis media (AOM) [2,3].

In Latin America and the Caribbean, Spn is estimated to account for 18,000 pneumococcal deaths, 327,000 cases of pneumonia, 4,000 meningitis and 1,229 sepsis each year in children aged under five years [2]. In addition, around 10.5 million episodes of AOM occur in children under 5 years of age per annual birth cohort [2,4], 30% of which are caused by Spn [5] and 18% per *H. influenza*[5]. The overall burden of pneumococcal disease in the pediatric population in the region is estimated at 600,000 Disability Adjusted Life Years [2]. However, the incidence and mortality of pneumococcal diseases vary across the countries [3].

New conjugate vaccines have been developed that are immunogenic in children younger than 2 years of age. A recently developed 10-valent pneumococcal non-typable *H influenzae* protein D-conjugate vaccine (PHiD-CV) offers a broad spectrum of coverage against Spn strains including serotypes 1, 4, 5, 6B, 7F, 9V, 14, 18C, 19F and 23F. Some of these serotypes have a high prevalence in Latin America and the Caribbean. Serotype 14 accounts for 21.1% of all invasive isolates in the region (29.1% in children under 6 years old) [6]. According to this profile, PHiD-CV is expected to afford protection against 76.5% of isolates causing invasive pneumococcal disease (IPD) in children aged < 5 years in Latin America [6]. Moreover PHiD-CV offers additional benefits since 8 of 10 vaccine strains are conjugated to protein D from non-typable *Haemophilus influenzae* (NTHi) and there is evidence that the vaccine induces additional protection against acute otitis media caused by both Spn and NTHi [7].

The introduction of pneumococcal vaccination in regional routine infant immunization programs may have a highly significant impact on health budgets, particularly in developing countries where health care resources are limited. However, several other factors constrain the incorporation of new vaccines into local immunization programs [8]. The availability of accurate and reliable data therefore becomes crucial to foster decision-making based on cost effectiveness criteria. Even though initial healthcare costs may be high, delayed vaccine introduction may result in thousands of immuno-preventable disease and deaths and many years of healthy life lost. Cost- effectiveness analysis is a useful tool for assessing issues of efficiency in health care resource allocation. By comparing economic evaluation results with country-specific willingness to pay thresholds, decision makers can better evaluate cost-effectiveness of health interventions at a national level.

The aim of this study was to assess the cost-effectiveness of PHiD-CV in comparison to no vaccination in children under 10 years of age in six Latin American countries: Argentina, Brazil, Chile, Colombia, Mexico and Peru. In these countries, pharmacoeconomic data were required formally or informally to facilitate the decision making process of vaccine introduction and this paper was generated to address this need.

## Material and methods

### Model

We used a previously described static, deterministic decision tree simulation model [9]. The model was adapted to estimate the health burden and costs related to pneumococcal disease in the population under 10 years old and to determine cost-effectiveness of PHiD-CV, during one year and assuming steady state. This steady state reflects the outcomes associated with vaccination of a cohort of newborns in the current year, as well as the carry-over effects of vaccination from previous years of older cohorts. Net present value of all future costs and benefits (during each person’s expected lifetime) is imputed to the steady-state population during the current year. The model used a deterministic framework to assess the impact of vaccination on the population by calculating the reduction in the incidence of disease associated with Spn and NTHi and estimated both vaccination program costs as well as total costs averted with vaccination. Four different main health outcomes were included in the model: all-cause pneumonia (hospitalized and ambulatory), all-cause AOM (including tube insertion), pneumococcal meningitis and pneumococcal bacteremia. Only cases of hospitalized IPD, which encompasses meningitis and bacteremia, were considered. Chronic hearing sequelae after AOM and chronic hearing and neurologic sequelae after meningitis were also assessed. Model framework and health events addressed were previously described in detail [9].

The model included two different strategies: no vaccination and vaccination with PHiD-CV. We assumed vaccination coverage reached 95% of the target population. This percentage was considered adequate to estimate current national immunization coverage levels in most countries included in the analysis, as its value is similar to actual coverage rates [10]. The dosing regimen considered consists in three vaccine doses (at 2 months, 4 months and 6 months) and a booster dose (at 13 months) (3 + 1 schedule). The vaccine is considered to provide some level of protection from the first dose through the assumed duration of efficacy [9]. For the main analysis only direct effects of vaccination in children under the age of 10 years old were considered, assuming no protection against pneumococcal disease in older children and adults. As the herd immunity varies across countries with different effects, a secondary analysis [11-14] estimated indirect effects of vaccination on the population (herd immunity and serotype replacement). Principal inputs to the model included epidemiological information on disease incidence, disutility and costs associated with disease events, vaccine effectiveness and costs of vaccination. Model outcomes included number of cases prevented, deaths averted, quality-adjusted life- years (QALYs) gained and costs.

### Epidemiological data

Local epidemiological data were incorporated for each country to reflect country-specific context. Data were retrieved from different sources. First, we conducted two systematic reviews (bibliographic databases MEDLINE, EMBASE, LILACS, regional scientific meeting abstracts, national statistics and statistics from international organizations) in order to determine incidence, etiology and use of health resources for pneumonia and AOM in the pediatric population in Latin America and the Caribbean region [5,15]. An additional literature review was conducted to obtain information not contained in prior systematic reviews for IPD. Local key opinion leaders in pediatrics infectious diseases were asked to provide bibliographic sources when available. Second, for data not found in published literature, we sought expert consensus using country level modified Delphi method [16]. Two expert panels were convened in each country, one for ambulatory and the other for hospitalized health events. Each panel comprised 6 to 8 local pediatric-oriented experts who participated in two rounds of questions by mail. The selection of the physicians that accepted to participate were a convenience sample representative of the different health system subsector.

After the model was completed, epidemiological input parameters and burden of disease estimations obtained for each country were validated via review with local experts. Demographic estimates for 2008 grouped by age and gender were used for each country to convert rates into absolute numbers as required by the model [17-21]. In the case of Peru and Argentina, demographic estimates used corresponded to 2007 and 2006 respectively [22,23].

### Baseline disease estimations

Burden of disease for the different health events was estimated taking into account the following inputs that were included in the model: hospitalization rates, case fatality ratios (CFRs), hospitalization ratios, and the incidence of ambulatory cases.

For all-cause pneumonia, hospitalization rates and CFRs for Brazil, Chile and Mexico were obtained from national health statistics: Sistema Único de Saúde (SUS) in Brazil [24], National Health System in Chile (División de Planificación Sanitaria. Departamento de Estadísticas e Información de Salud. Ministerio de Salud de Chile, personal communication. April, 2009), and from the social security Instituto Mexicano del Seguro Social in Mexico (IMSS) [25]. As hospitalization rates were not available for Colombia, Peru and Argentina, estimates of all cause pneumonia were derived from mortality rates using Equation 1. In the case of Colombia and Peru mean CFR for Brazil, Chile and Mexico were applied, and it was obtained from Delphi Panel in the case of Argentina. In all countries we assumed that all deaths occurred during hospitalization, except for Peru, where only 46% of deaths were assumed to occur in hospital [26].

(1) $$\begin{array}{l} \mathrm{Incidence}\;\mathrm{of}\;\mathrm{hospitalized}\;\mathrm{pneumonias}\\ \;=\frac{\mathrm{pneumonia}\;\mathrm{mortality}\;\mathrm{rate}}{\mathrm{case}\;\mathrm{Fatality}\;\mathrm{Ratio}} \end{array}$$

Ambulatory pneumonia cases were estimated in all countries from hospitalization rates based on the proportion of pneumonia requiring hospitalization (*hospitalization ratio*) provided by Delphi panels, using Equation 2.

(2) $$\begin{array}{l} \mathrm{Incidence}\;\mathrm{of}\;\mathrm{ambulatory}\;\mathrm{pneumonias}\\ \;=\frac{\mathrm{incidence}\;\mathrm{of}\;\mathrm{hospitalized}\;\mathrm{pneumonias}}{\mathrm{hospitalization}\;\mathrm{ratio}}\\ \;–\;\mathrm{incidence}\;\mathrm{of}\;\mathrm{hospitalized}\;\mathrm{pneumonias} \end{array}$$

The number of ambulatory cases of all-cause AOM for Brazil and Mexico was derived from local studies [27,28]. Incidence rate of AOM in children under 4 years-old from Chile was obtained from a local study [29], assuming a decrement in incidence after that age similar to that reported in Brazil. Incidence rate for Colombia, Peru and Argentina was assumed to be the mean rate of data from Brazil, Chile and Mexico. In addition, for all countries the number of myringotomy procedures due to all-cause AOM and the percentage of cases of AOM-related hearing sequelae were calculated by multiplying AOM incidence by the corresponding percentages provided by the Delphi panels.

For IPD, only hospitalized cases were included. The incidence of pneumococcal meningitis and pneumococcal bacteremia were estimated separately based on the incidence of all-cause IPD and the percentage of cases caused by Spn using Equation 3. Incidence of all-cause IPD for Brazil, Chile and Mexico were obtained from same local data sources mentioned for pneumonia. Values were derived from mortality rates using Equation 1 for Colombia, Peru and Argentina. Percentage of IPD cases attributable to pneumococci, CFRs for IPD and percentage of meningitis-related hearing and neurologic sequelae were derived from Delphi panels in all countries.

(3) $$\begin{array}{l} \mathrm{Incidence}\;\mathrm{of}\;\mathrm{Spn}\;\mathrm{invasive}\;\mathrm{disease}\;\left(\mathrm{ID}\right)\\ \;=\mathrm{Incidence}\;\mathrm{of}\;\mathrm{ID}\;\mathrm{x \%}\;\mathrm{of}\;\mathrm{ID}\;\mathrm{associate}\;\mathrm{to}\;\mathrm{Spn} \end{array}$$

Hospitalization rates for the different diseases were extrapolated from national statistics, after adjusting for estimated population coverage. Data were obtained from the IMSS database for Mexico [19] assuming 42.2% of the population had access to social security coverage [25]. Data were collected from DATASUS database for Brazil [30], assuming 87% coverage, (similar to mortality data [31]). Data for Chile were provided by the Department of Health Statistics and Information (DEIS) (División de Planificación Sanitaria 2009 Departamento de Estadísticas e Información de Salud. Ministerio de Salud de Chile. Personal Communication) assuming 94% coverage according to the World Health Organization’s Statistical Information System (WHOSIS) [32]. Hospitalization rates for pneumonia, meningitis and bacteremia for Colombia were extrapolated from mortality rates obtained from the National Administrative Department of Statistics (DANE) adjusted for estimated coverage of mortality collected from WHOSIS [18,32] assuming 81% coverage. Similarly, hospitalization rates for Peru were extrapolated from mortality rates provided by the General Office of Statistics and Information (OGEI) [33] assuming 68% coverage according to WHOSIS [32]. In the case of Argentina, mortality rates were obtained from Health Statistics and Information Department (DEIS) [22] assuming 94% coverage according to WHOSIS [32]. Tables 1 and 2 summarize the epidemiological input data included in the models.

**Table 1:** Epidemiological input data: acute events

	**Argentina**		**Brazil**		**Chile**		**Colombia**		**Mexico**		**Peru**	
	Incidence rate*	**CFR† (%)**	Incidence rate*	**CFR† (%)**	Incidence rate*	**CFR† (%)**	Incidence rate*	**CFR† (%)**	Incidence rate*	**CFR† (%)**	Incidence rate*	**CFR† (%)**
All-cause pneumonia (hospitalized) ‡												
0-1 years	1,323.80	3.5	4,026.60	0.8	6,526.20	0.5	2,715.30	3.4	639.8	8.9	2,130.50	7.4
1-4 years	257.4	1.5	1,562.80	0.2	1,563.20	0.1	1,147.76	0.8	273.31	2	1,034	1.7
5-9 years	109.9	1	362	0.2	388.6	0	273.3	0.6	31.2	1.6	262.6	1.4
All-cause pneumonia (ambulatory) §												
0-1 years	441.3	-	3,643.10	-	15,227.80	-	301.7	-	185.8	-	236.7	-
1-4 years	922.4	-	4,748.40	-	7,555.30	-	1,265.48	-	956.6	-	646.24	-
5-9 years	1,721.90	-	3,775.10	-	7,384.30	-	2,211	-	281	-	1,050.40	-
All-cause AOM ll												
0-1 years	8,943.50	-	6,050.90	-	16,260.20	-	8,943.50	-	4,519.40	-	8,943.50	-
1-4 years	7,690.50	-	7,563.60	-	10,988.60	-	7,690.55	-	4,519.40	-	7,690.50	-
5-9 years	4,626.90	-	2,915.30	-	2,882.60	-	4,626.90	-	1,921.90	-	4,626.90	-
Pneumococcal meningitis¶												
0-1 years	19.4	16.1	22.5	40	39.4	12.3	28.4	17.5	5.76	40	5.1	20
1-4 years	0.74	16.1	4.6	25	5.4	10	5.35	13.75	0.61	18.5	1.62	7.5
5-9 years	0.12	16.1	2.3	10	3.1	10	1.4	4	0.07	5.5	4.9	2
Pneumococcal bacteraemia/sepsis**												
0-1 years	54.2	5.5	171.2	22.5	61.5	7.5	61.7	12.5	101.8	30	54.2	20
1-4 years	9.16	4	8.9	11.3	6.8	2	9.3	7.5	8.94	17.5	8.52	12.5
5-9 years	4.33	1	2	6.5	1.3	2	2.1	3.5	0.42	5	2.5	2

* per 100,000 people.

†*CFR* = Case Fatality Ratio.

‡Pneumonia hospitalization rate and CFR for Brazil and Chile were obtained from national health statistics: Sistema Único de Saúde (SUS) in Brazil [24], National Health System in Chile (División de Planificación Sanitaria. Departamento de Estadísticas e Información de Salud. Ministerio de Salud de Chile, personal communication. April, 2009); The same figures for Mexico were obtained from the social security Instituto Mexicano del Seguro Social (IMSS) [25]. Estimates for Colombia, Peru, and Argentina were derived from mortality rates applying mean CFR for Brazil, Chile and Mexico. In all countries we assumed that all deaths occurred during hospitalization, except Peru, where only 46% of deaths were assumed to occur in the hospital [26].

§Cases of ambulatory pneumonia were estimated in all countries from hospitalization rates based on the proportion of pneumonia requiring hospitalization provided by Delphi panels.

ll Number of cases of AOM for Brazil and Mexico were derived from local studies [27,28]. Incidence rate for children under 4 years-old was obtained from other local study for Chile [29]., assuming the same age decrement in the incidence rate as described for Brazil Incidence rates for Colombia and Peru were assumed to be the mean rate between Brazil, Chile and Mexico.

¶ ** The incidence of pneumococcal meningitis and of pneumococcal bacteremia were estimated separately based on the incidence of all-cause IPD and the percentage of cases caused by Spn. Incidence of all-cause IPD for Brazil, Chile and Mexico were obtained from the same sources as pneumonia. Incidence of all-cause IPD for Colombia, Peru and Argentina were derived from mortality rates. Percentage of IPD cases attributable to Spn and CFRs for IPD were derived from Delphi panels for all countries. Delphi panels in Brazil were provided with additional information from local studies.

**Table 2:** **Epidemiological input data: complications and chronic sequelae**^**1**^

	**Argentina**			**Brazil**			**Chile**			**Colombia**			**Mexico**			**Peru**		
	**Myringot.**	**Hearing seq**	**Neurol seq**	**Myringot.**	**Hearing seq**	**Neurol seq**	**Myringot.**	**Hearing seq**	**Neurol seq**	**Myringot.**	**Hearing seq**	**Neurol seq**	**Myringot.**	**Hearing seq**	**Neurol seq**	**Myringot.**	**Hearing seq**	**Neurol seq**
All-cause AOM																		
0-1 years	0.02%	10.0%	-	0.05%	10%	-	0.03%	10%	-	0.07%	10%	-	0.02%	12.50%	-	0.01%	13.50%	-
2-4 years	0.02%	4.00%	-	0.02%	4%	-	0.03%	4.50%	-	0.07%	7.50%	-	0.05%	7.50%	-	0.04%	6.24%	-
5-9 years	0.01%	2.00%	-	0.01%	1%	-	0.02%	2.50%	-	0.01%	2%	-	0.03%	3%	-	0.01%	1.50%	-
Pneumococcal meningitis																		
0-1 years	-	5.88%	17.63%	-	10%	40%	-	25%	25%	-	56.30%	18.80%	-	12%	28%	-	6%	24%
2-4 years	-	2.34%	14.53%	-	10%	40%	-	11.10%	20.10%	-	25.50%	23.25%	-	4.25%	17%	-	4.30%	23.25%
5-10 years	-	0.70%	9.30%	-	10%	36%	-	5%	15%	-	58.20%	15.28%	-	1.90%	10.60%	-	0.50%	9.50%

^1^All these data were obtained from Delphi panels.

### Vaccine effectiveness

Since evidence of vaccine effectiveness against pneumonia is not available for PHiD-CV at the moment of the analysis, it was estimated using data from the Northern California Kaiser Permanente trial for the 7-valent conjugate vaccine (PCV-7) which contains seven shared serotypes [34] and assumed to be the same for all participating countries (Table 3). Vaccine effectiveness for PHiD-CV against non hospitalized pneumonia was assumed to be 4.3% as it was demonstrated against clinical pneumonia [34] for PCV-7, and used as a proxy for non hospitalized pneumonia. PHiD-CV was assumed to be slightly more effective (25% effectiveness instead of 20.5% as shown in NCKP trial [34]) than PCV-7 against radiologically confirmed pneumonia (used as a proxy for hospitalized pneumonia) based on expert opinion given that the additional serotypes covered by this vaccine are frequent agents of severe pneumonia.

**Table 3:** Maximum vaccine effectiveness

	**Argentina**	**Brazil**	**Chile**	**Colombia**	**Mexico**	**Peru**
		**PHiD-CV**				
All-cause pneumonia (hospitalized)	25%	25%	25%	25%	25%	25%
All-cause pneumonia (ambulatory)	4.30%	4.30%	4.30%	4.30%	4.30%	4.30%
All-cause AOM*	29.90%	18%	24.60%	18.20%	20.70%	18.20%
Myringotomy procedure	85.80%	51.72%	70.53%	52.13%	59.55%	52.13%
IPD†	76.29%	77.82%	71.60%	80.41%	67.73%	69.99%

*AOM Acute otitis media.

†IPD Invasive pneumococcal disease.

In the case of AOM, vaccine effectiveness was calculated based on vaccine efficacy and Spn and NTHi prevalence. The model used the distribution of Spn serotypes and NTHi pathogens to calculate the percentage of cases avoided. Because vaccine effectiveness against AOM was not available by serotype, estimation was based on efficacy against episodes due to pneumococcal types covered by the vaccine, against episodes due to non-vaccine serotypes and against AOM episodes caused by NTHi (Table 3). Efficacy data for PHiD-CV were taken from an 11-valent version of PHiD-CV study by Prymula et al. [7], showing a 58% and 36% efficacy to reduce vaccine covered pneumococcal serotypes AOM and NTHi episodes respectively. Although not observed in Prymula et al. study, an increase in the frequency of AOM due to non-covered pneumococcal serotypes (type replacement) was assumed for PHID-CV, as described for PCV-7 (33% increase) based on Eskola et al. [35] and expert recommendation. In addition, cross protection against AOM due to pneumococcal serotype 6A of 76% was assumed [35,36]. Finally, maximum vaccine effectiveness was estimated as shown in Equation 4 considering the efficacy and prevalence for the three different groups of AOM pathogens. Local distribution of pneumococcal serotypes and NTHi were obtained from a meta-analysis previously cited [5,15]. In Argentina, Chile and Mexico specific sources identified by systematic review were also consulted [37-39].

(4) $$\begin{array}{ll} \;\mathrm{Effectiveness}= & \;\mathrm{\%Spn}\times \mathrm{ESpn}\;+\mathrm{\%Sp}n_{\mathrm{NC}}\\ & \times \mathrm{ESp}n_{\mathrm{NC}}+\mathrm{\%NTHi}\times \mathrm{ENTHi} \end{array}$$

Finally, vaccine effectiveness for reduction in myringotomy procedures was estimated by using data taken from Black et al. [40], showing that PCV-7 was about 2.87 times more effective in preventing myringotomies than AOM episodes. PHiD-CV was assumed to have the same relative effect than PCV-7 in reduction in myringotomies.

In order to estimate vaccine effectiveness against IPD, vaccine efficacy was adjusted for differences in pneumococcal serotype distribution. Vaccine effectiveness against IPD was based on serotype-specific efficacy from a case–control study reported by Whitney et al. [36] for PCV-7. Equal serotype-specific efficacy was assumed for PHiD-CV as described for the shared vaccine serotypes on PCV7. The average vaccine efficacy observed against PCV-7 vaccine serotypes (94.7%) was used for the additional serotypes covered by PHiD-CV, based on expert recommendation. Vaccine efficacy for IPD was adjusted using country-specific serotype distribution provided by PAHO’s Network System for the Surveillance of Bacterial Agents responsible for Pneumonia and Meningitis (SIREVA) [41,42] (Table 3). In addition, cross protection against 6A and 19A serotypes was assumed for IPD, based on cross protection demonstrated in PCV-7 studies [35,36,43] a review on cross protection to 19A [44] and cross reaction demonstrated in PHiD-CV immunogenicity studies [45].

Final vaccine effectiveness estimates were used to calculate the maximum reduction in disease among the vaccinated population (Table 3). These estimates represent the maximum effectiveness achieved by vaccination. The model assumes that there is a period of increasing efficacy, where the vaccine confers partial protection until the booster dose is administered. Full protective efficacy from the vaccine (maximum published efficacies) is assumed to begin after the booster dose. Because immunity may wane with time, the model assumes that the full protection provided after booster dose persists up to 3 years of age. Thereafter, full protection from the vaccine is assumed to decline lineally by 28% each year, up to the age of 10 years, when any direct protection is assumed to disappear, as previously detailed [9].

No herd immunity was considered in the base case scenario. There is no clear pattern in herd effect observed across countries following the introduction of PCV7 for the reduction of invasive disease [11,12,14]. To date, the magnitude of the net indirect effect in the US has not been clearly replicated in other settings with high PCV7 use. Due to the lack of a clear herd effect process and the fact that the net indirect effect result may be a consequence of many different factors (e.g. vaccine and non-vaccine serotype coverage, duration of national immunization, epidemiology, vaccine schedule, immunization coverage, force of infection, contact matrix and demography), no herd immunity was considered in the base case scenario to produce a conservative analysis. In the scenarios where herd immunity was evaluated, we used a fixed herd effect value. In this scenario only herd protection against invasive disease was modeled. A net reduction of 15.4% was assumed in children under five and a net reduction of 29% was assumed from five years old until death [46].

### Costs

Both direct and indirect costs were included in the model. Direct costs comprised: vaccine cost and cost of treatment for pneumococcal diseases and sequelae, and are reported in 2008 US Dollars (US$) for Brazil, Chile, Colombia, México and 2009 US$ for Argentina and Peru. The differences in cost years in some of the countries are due to the date of availability of costs when they were obtained and also we tried to match the epidemiological data years. They were not inflationed or deflated to a common country date due to the potential error that it can be introduced with this methodology because of particular characteristics of regional economies [47]. Given that costs for acute conditions are incurred in the same year as that of the analysis, no discount was applied to them. However, an annual 3.5% discount was applied to future costs associated to sequelae or chronic consequences.

We estimated country specific costs for pneumococcal disease and for sequelae. Micro-costing method was used which permits use of detailed resource categories as well as utilization rates and unit prices for each health event. In all countries utilization rates for each event were estimated by Delphi method. Unit costs reported by SOAT [48] and by IMSS [49] were selected for resources used for health events and hearing sequelae (hearing aids and cochlear implants) for Colombia and Mexico, respectively. In Chile costs for resources were obtained from CIGES 2008 (Centro de Capacitación, Investigación y Gestión para la Salud Basada en Evidencias (2008) Ministerio de Salud de Chile) and (Temuco: Universidad de La Frontera. Unpublished data) and unit costs for hearing sequelae were obtained from Garantías Explícitas en Salud [50]. In Brazil, unit costs for health event resources were provided by SIA/SUS and DATASUS [24,51-53] and for hearing sequelae by DATASUS. However, for events requiring hospitalizations in Brazil, final costs were estimated by macro-costing method (DATASUS source) [24]. In Peru, costs were estimated as an average from three different sources, representing public [54], social security (from Sub Gerencia de Costos del Seguro Social de Salud, personal communication) and private sectors (from Complejo Hospitalario San Pablo, personal communication). The same approach was used for Argentina, taking into account the proportion of persons in each subsector [55].

Table 4 shows the resulting costs for the events included in the model. Vaccination costs included those corresponding to unit dose cost, average number of doses, coverage rates, supplies and human resources. Cost of 20 US$ per dose was used, assuming 10% losses from waste and 1 US$ cost of administration according to Constenla et al. [56].

**Table 4:** Cost per event

**Disease Outcome**	**Argentina**		**Brazil**		**Colombia**		**Chile**		**Mexico**		**Peru**	
	**Children**	**Adults**	**Children**	**Adults**	**Children**	**Adults**	**Children**	**Adults**	**Children**	**Adults**	**Children**	**Adults**
**Average cost per acute episode**												
Meningitis - hospitalized	$2,166	$8,959	$548	$548	$1,407	$1,251	$2,308	$2,432	$15,484	$11,767	$506	$489
Bacteremia - hospitalized	$1,002	$2,309	$417	$417	$1,090	$1,081	$578	$1,680	$6,444	$6,623	$253	$390
Pneumonia - hospitalized	$1,357	$1,930	$401	$401	$529	$669	$671	$864	$4,437	$6,828	$198	$303
Pneumonia - ambulatory	$127	$134	$32	$33	$52	$44	$62	$72	$427	$462	$31	$48
AOM hospitalized myringotomy	$57	$97	$214	$214	$56	$40	$32	$50	$476	$272	$25	$27
AOM GP† consultations	$68	$81	$24	$12	$31	$27	$35	$33	$343	$231	$20	$19
**Annual cost for long-term sequelae**												
Meningitis sequelae‡	$215	$102	$430	$543	$400	$112	$217	$59	$2,694	$856	$312	$83
Hearing loss 1st year	$526	$474	$220	$243	$212	$193	$151	$193	$254	$378	$18	$78
Hearing loss long term follow up	$152	$101	$34	$57	$92	$74	$140	$182	$203	$327	$18	$78

All costs are expressed in U$S Dollars. In Brazil, Colombia, Chile and México 2008 local costs were converted to US$ and in Argentina and Perú 2009 local costs were converted to US$ exchange rates are provided in methods.

†*GP*, General practitioner.

‡except hearing loss.

Indirect costs were estimated based on loss of productivity due to premature mortality from pneumococcal related diseases and costs incurred by family members caring for sick children. To assess the former, current net value of expected future earnings according to age at time of death was estimated (accumulating at a yearly discount rate of 3.5%); and for the latter, indirect costs were calculated based on human capital approach for one parent during the acute event (in which case no discount applies given that losses fall within the year of analysis) based on mean wage salaries for each country [55,57-61].

### Utilities

Normative utilities and disutilities values used in the model were obtained from international sources due to the lack of local data (see Table 5 for values used and references) and then used to estimate population Quality-Adjusted Life-Years (QALYs) loss. Losses in future utilities due to premature death or long term sequelae associated to events occurring during the study year were assigned to the present year at an annual discount rate of 3.5% in order to estimate the net present value. Life expectancy was estimated using normative utilities according to age [62].

**Table 5:** Yearly disutilities according to condition

	**Disutility value**	**Source**
*Short term disutilities associated with acute pathologies†*		
Pneumonia (hospitalized)	0.008	Assumed the same as for hospitalized bacteremia [63]
Pneumonia (ambulatory)	0.006	[63]
AOM (ambulatory)	0.005	[64]
AOM with myringotomy	0.005	Assumed the same as for AOM without myringotomy
Pneumococcal meningitis	0.023	[63]
Pneumococcal bacteremia	0.008	[63]
*Long term disutilities associated with sequelae‡*		
Hearing loss due to AOM	0.090	[65]
Neurologic sequelae due to meningitis	0.400	[66]
Hearing loss due to meningitis	0.200	[66]

*AOM,* Acute Otitis Media.

†Applied to current year without discount.

‡Applied to current and subsequent years with discount.

### Cost-effectiveness

Comparison of health and economic results for PHiD-CV vaccination compared to no vaccination, allowed us to determine differences in health benefits and costs attributable to vaccination. Based on these results, and in the case where vaccination was more costly as well as more effective, incremental cost-effectiveness ratio (ICER) per QALY saved and Life Year gained was estimated, representing additional costs per year of life gained adjusted for quality.

The main analysis was performed from a payer’s perspective, in which only direct costs were considered. For this analysis, we used as a decision rule the Commission on Macroeconomics and Health recommendations, which define as very cost effective those interventions costing less than one time the Gross Domestic Product (GDP) per capita for each disability adjusted life year (DALY) averted, and as cost-effective those interventions costing less than three times the GDP per capita for each DALY averted. These thresholds are adhered to by the WHO [67]. For our main analysis, we considered a cost-effectiveness threshold of less than three times 2008 GDP per capita for Brazil R$ 45,781.53 Reais [68]; Chile $15,894,820.09 Chilean Pesos [68]; Colombia $ 29,790,816.26 Colombian Pesos [68] and Mexico $ 341,576.84 Mexican Pesos [68]. The threshold was based on 2009 GDP per capita $ 40,107.46 Nuevos Soles for Peru [68], and on 2009 GDP per capita $ 86,425.31 Argentine Pesos for Argentina [68]. 3xGDPs are expressed in US$ Dollars with the following rates [68], for Argentina we used 2009 GDP per capita ($ = Argentinean pesos) 1US$ = 3.87 2009 Argentina pesos, for Brazil we used 2008 GDP per capita ($ = Reais) 1US$ = 1.84 2008 Reais, for Colombia we used 2008 GDP ($ = Colombian pesos) 1US$ = 1,990 2008 Colombian pesos, for Mexico we used 2008 GDP ($ = Mexican pesos) 1US$ = 11.16 2008 mexican pesos and for Peru we used 2009 GDP ($ = Nuevo soles) 1US$ = 3.05 2009 Nuevos soles. Also cost-effectiveness ratios are reported in International Dollars with the following rates, for Argentina we used 1 I$ = 2 Argentine pesos, for Brazil we used 1 I$ = 1.6 Reais, for Chile we used 1 I$ = 376.1 Chilean pesos, for Colombia we used 1 I$ = 1,228 Colombian pesos, for Mexico we used 1 I$ = 8.1 Mexican pesos and for Peru we used 1 I$ = 1.5 Nuevos soles [68].

### Sensitivity analysis

We performed one-way sensitivity analyses to explore the impact of different parameters included in the model on study results (comparing PHiD-CV vaccination strategy against non-vaccination). Each parameter was varied between a range of values up and down from the base case value. For each change, the ICER was re-estimated and compared to the selected cost-effectiveness threshold. Results were considered robust when ICER variations were not sufficient to modify interpretation of cost-effectiveness of pneumococcal immunization. Table 6 shows the parameters included, the base case values and the parameters ranges used in the sensitivity analyses.

**Table 6:** Parameters included in sensitivity analysis comparing PHiD-CV vaccination strategy against non- vaccination

**Variable**	**Base case**	**Range of sensitivity analysis**
Pneumonia – Incidence	Age-specific data	-/+20% for hospitalizations
		-/+50% for ambulatory cases
Pneumonia – Case fatality rate	Age-specific data	-/+20%
AOM – Incidence	Age-specific data	-/+20% for myringotomies
		-/+50% for total cases
AOM - Etiology	Country-specific data	-/+20% for Sp cases
		-/+40% for NTHi cases
AOM – Etiology	Country-specific data	-/+20% for SpC cases
Meningitis – Incidence	Age-specific data	-/+50%
Meningitis – Case fatality rate	Age-specific data	-/+20%
Meningitis – Risk of sequelae	Age-specific data	-/+20%
Bacteremia – Incidence	Age-specific data	-/+50%
Bacteremia – Case fatality rate	Age-specific data	-/+20%
ID - Etiology (Sp serotype distribution)	Age-specific data	95% CI
ID - Etiology (Sp in ≥10 years)	Age-specific data	-/+20%
Effectiveness in reducing hospitalizations for pneumonia	25%	95% CI
Effectiveness in reducing ambulatory pneumonia	4,3%	95% CI
Efficacy in preventing AOM according to type of pathogen (Sp_C_, Sp_NC_ y HiNT)	57.6% for Sp_C_	95% CI
	-33% for Sp_NC_	
	35.3% for NTHi	
Efficacy in preventing ID	Based on efficacy by serotype for PCV-7	95%CI for each serotype
Costs of administration and vaccine wastage*	1 dollar with 10% wastage	-/+20%
Direct cost of treatment of acute events	Disease-specific data	-/+20%
Annual cost of long term treatment of sequelae	Data specific for each condition	-/+20%
Disutilities associated with AOM (with and without myringotomy)	Data specific for each condition	95% CI
†Disutilities associated with acute diseases except AOM†	Data specific for each condition	95% CI
Disutilities associated with chronic conditions (sequelae)‡	Data specific for each condition	95% CI

*ID*, Invasive Disease; *NTHi*, Non typable Haemophilus influenzae: 95% CI: 95% confidence interval; *AOM,* Acute Otitis Media; *Sp*, Streptococcus pneumoniae; *SpC,* Streptococcus pneumoniae serotypes covered by vaccine; *SpNC* Streptococcus pneumoniae serotypes not covered by vaccine.

†Per episode.

‡Per year.

## Results

The projected health outcomes without routine immunization with PHiD-CV are shown in Table 7. Table 8 summarizes the same results but with PHiD-CV vaccination. All the results and estimations are from birth till 10 years old. The number of cases prevented in the current year is provided for each country. The number of deaths avoided and the total number of life years and QALYs gained are also shown. Vaccination with PHiD-CV caused an important reduction in number of disease events for all health outcomes.

**Table 7:** Health outcomes and costs without vaccination^*^

**Health outcomes (number of cases)**	**Argentina**	**Brazil**	**Chile**	**Colombia**	**Mexico**	**Peru**
Pneumonia hospitalizations	19 435	438 803	36 761	78 137	36 925	40 710
Ambulatory pneumonia	86 086	1 487 153	207 167	148 821	108 124	43 833
Total pneumonia	105 521	1 925 956	243 928	226 958	145 049	84 543
Myringotomies	7088	36 059	5229	28 769	25 516	8072
AOM sequelae	16 005	12 490	29 490	22 448	13 395	24 362
Total AOM	422 134	1 834 559	186 538	567 084	645 551	339 758
Pneumococcal meningitis sequelae	29	666	65.5	248	36	28
Total pneumococcal meningitis	153	1913	191.9	511	166	194
Pneumococcal bacteremia	753	7927	237	989	2702	528
**Health outcomes (deaths)**						
Pneumonia (% from total)	449 (89.26%)	1843 (46.32%)	103 (75.18%)	1234 (88.08%)	1575 (67.34%)	1271 (93.39%)
Pneumococcal meningitis (% from total)	25 (4.97%)	552 (13.87%)	21.4 (15.62%)	69 (4.93%)	53 (2.27%)	10 (0.73%)
Pneumococcal bacteremia (% from total)	29 (5.77%)	1584 (39.81%)	13 (9.49%)	97 (6.92%)	711 (30.40%)	80 (5.88%)
Total deaths	503	3979	137	1401	2339	1361
**Life-years lost due to premature death**						
Pneumonia (% from total)	11 955 (89.32%)	47 835 (46.38%)	2767 (74.82%)	32 442 (88.10%)	41 797 (67.34%)	33 310 (93.37%)
Pneumococcal meningitis (% from total)	656 (4.90%)	14 336 (13.90%)	576 (15.58%)	1822 (4.95%)	1400 (2.26%)	271 (0.76%)
Pneumococcal bacteremia (% from total)	775 (5.79%)	40 974 (39.73%)	355 (9.60%)	2560 (6.95%)	18 869 (30.40%)	2096 (5.87%)
Total life- years lost due to premature death	13 385	103 144	3698	36 823	62 066	35 677
**QALYs lost**						
Pneumonia (% from total)	11 319 (21.19%)	54 930 (36.80%)	3979 (5.10%)	30 423 (32.92%)	38 200 (41.48%)	30 291 (32.90%)
AOM (% from total)	40 537 (75.80%)	38 632 (25.88%)	72 612 (93.12%)	56 304 (60.92%)	35 453 (38.50%)	59 385 (64.50%)
Pneumococcal meningitis (% from total)	857 (1.60%)	19 050 (12.76%)	1067 (1.37%)	3400 (3.68%)	1583 (1.72%)	525 (0.57%)
Pneumococcal bacteremia (% from total)	697 (1.30%)	36 656 (24.56%)	318 (0.41%)	2291 (2.48%)	16 853 (18.30%)	1874 (2.04%)
Total QALYs lost	53 410	149 267	77 976	92 418	92 089	92 075
**Direct costs**						
Pneumonia (% from total)	$ 37 285 729 (28.04%)	$ 223 194 337 (75.27%)	$ 37 536 647 (23.94%)	$ 48 992 398 (38.69%)	$ 209 948 462 (39.02%)	$ 9 452 263 (33.30%)
AOM (% from total)	$ 94 445 835 (71.03%)	$ 62 909 086 (21.22%)	$ 118 366 250 (75.49%)	$ 74 526 550 (58.85%)	$ 306 229 730 (56.91%)	$ 18 495 287 (65.16%)
Pneumococcal meningitis (% from total)	$ 485 136 (0.36%)	$ 7 110 442 (2.40%)	$ 766 387 (0.49%)	$ 2 036 356 (1.61%)	$ 4 508 280 (0.84%)	$ 303 882 (1.07%)
Pneumococcal bacteremia (% from total)	$ 754 814 (0.57%)	$ 3 307 190 (1.12%)	$ 137 010 (0.09%)	$ 1 077 341 (0.85%)	$ 17 412 377 (3.24%)	$ 133 771 (0.47%)
Total direct costs	$132 971 513	$296 521 055	$156 806 295	$126 632 644	$ 538 098 849	$ 28 385 203
**Indirect costs**						
Pneumonia (% from total)	$ 26 513 765 (26.15%)	$ 205 729 961 (46.78%)	$ 15 989 127 (10.53%)	$ 57 757 864 (39.05%)	$ 55 732 299 (38.26%)	$ 49 187 200 (36.56%)
AOM (% from total)	$ 71 941 758 (70.95%)	$ 77 923 319 (17.72%)	$134 513 520 (88.57%)	$ 82 516 905 (55.78%)	$ 43 309 768 (29.73%)	$ 79 622 249 (59.18%)
Pneumococcal meningitis (% from total)	$ 1 473 497 (1.45%)	$ 78 064 982 (17.75%)	$ 681 723 (0.45%)	$ 3 823 700 (2.58%)	$ 23 319 138 (16.01%)	$ 2 864 722 (2.13%)
Pneumococcal bacteremia (% from total)	$ 1 473 497 (1.45%)	$ 78 064 982 (17.75%)	$ 681 723 (0.45%)	$ 3 823 700 (2.58%)	$ 23 319 138 (16.01%)	$ 2 864 722 (2.13%)
Total indirect costs	$101 402 517	$439 783 244	$151 866 092	$147 922 168	$145 680 345	$134 538 893

*Analysis is from birth till 10 years of age, discount rate 3.5. All costs are expressed in U$S Dollars. In Brazil, Colombia, Chile and México 2008 local costs were converted to US$ and in Argentina and Perú 2009 local costs were converted to US$ exchange rates are provided in methods.

% from total refers to the percentage of cases attributable to the condition from the total of cases or costs.

**Table 8:** Health outcomes and costs after PHiD-CV vaccination: comparative analysis vs non-vaccination*

	**Argentina**	**Brazil**	**Chile**	**Colombia**	**Mexico**	**Peru**
**Health outcomes (events averted)**						
Pneumococcal hospitalizations - N(%)	2851 (14.67)	69 415 (15.82)	5709 (15.53)	12 257 (15.69)	6112 (16.55)	6391 (15.70)
Ambulatory pneumonia - N(%)	1292 (1.50)	30 443 (2.05)	4198 (2.03)	2270(1.53)	2887 (2.67)	689 (1.57)
Total pneumonia - N(%)	4143 (3.93)	99 859 (5.18)	9907 (4.06)	14 527 (6.40)	8999 (6.20)	7080 (8.37)
Myringotomies - N(%)	3708 (52.32)	12 532 (34.75)	2461 (47.06)	11 031 (38.34)	9395 (36.82)	2945 (36.48)
AOM sequelae - N(%)	3272 (20.45)	1643 (13.16)	5101 (17.30)	3085 (13.74)	2075 (15.49)	3209 (13.17)
Total AOM - N(%)	67 519 (15.99)	188 353 (10.27)	29180 (15.64)	55 098 (9.72)	75 534 (11.70)	33 323 (9.81)
Pneumococcal meningitis sequelae - N(%)	16 (56.65)	436 (65.46)	32 (48.96)	147 (59.36)	23 (64.87)	11 (37.80)
Pneumococcal meningitis - N(%)	72 (47.32)	876 (45.78)	78 (40.54)	243 (47.44)	70 (42.32)	52 (26.56)
Pneumococcal bacteremia - N(%)	319 (42.34)	3812 (48.08)	107 (45.07)	488 (49.34)	1169 (43.26)	232 (43.89)
**Health outcomes (deaths averted)**						
Pneumonoia - N(%)	69 (15.28)	284 (15.40)	16 (15.60)	192 (15.58)	251 (15.92)	199 (15.64)
Pneumococcal meningitis - N(%)	12 (47.32)	282 (51.06	9 (40.85)	36 (52.07)	23 (43.52)	41 (39.29)
Pneumococcal bacteremia - N(%)	14 (48.45)	764 (48.27)	6 (44.09)	50 (51.68)	306 (43.08)	37 (46.78)
Total deaths averted - N(%)	94 (18.77)	1330 (33.43)	31 (22.27)	279 (19.89)	580 (24.80)	240 (17.65)
**Life- years gained**						
Pneumonia - N(%)	1829 (15.30)	7379 (15.43)	432 (15.60)	5060 (15.60)	6660 (15.93)	5216 (15.66)
Pneumococcal meningitis - N(%)	310 (47.35)	7335 (51.17)	236 (40.95)	950 (52.13)	610 (43.55)	107 (39.39)
Pneumococcal bacteremia - N(%)	376 (48.50)	19 791 (48.30)	157 (44.11)	1324 (51.73)	8132 (43.10)	982 (46.84)
Total life- years gained - N(%)	2515 (18.79)	34 505 (33.45)	824 (22.28)	7334 (19.92)	15 402 (24.81)	6305 (17.67)
**QALYs gained**						
Pneumonia - N(%)	1661 (14.67)	7315 (13.32)	455 (11.43)	4623 (15.20)	6004 (15.72)	4708 (15.54)
AOM - N(%)	8215 (20.27)	4866 (12.60)	12 560 (17.30)	7671 (13.62)	5405 (15.25)	7789 (13.12)
Pneumococcal meningitis - N(%)	406 (47.42)	9336 (49.01)	449 (42.05)	1725 (50.72)	684 (43.23)	190 (36.13)
Pneumococcal bacteremia - N(%)	338 (48.45)	17 706 (48.30)	140 (44.11)	1185 (51.73)	7263 (43.10)	878 (46.83)
Total QALYs gained - N(%)	10658 (19.95)	39 223 (26.28)	13 604 (17.45)	15 204 (16.45)	19 357 (21.02)	13 565 (14.73)
**Direct costs (savings)**						
Pneumonia - N(%)	$ 4 033 877 (11)	$ 28 797 257 (13)	$ 4 092 194 (11)	$ 6 596 723 (13)	$ 28 348 389 (14)	$ 1 289 409 (14)
AOM - N(%)	$ 18 167 102 (19)	$ 8 683 587 (14)	$ 20 426 178 (17)	$ 9 909 792 (13)	$ 41 651 823 (14)	$ 2 252 166 (12)
Pneumococcal meningitis - N(%)	$ 229 857 (47)	$ 3 187 488 (45)	$ 319 239 (42)	$ 986 297 (48)	$ 1 902 325 (42)	$ 93 682 (31)
Pneumococcal bacteremia - N(%)	$ 319 575 (42)	$ 1 590 258 (48)	$ 61 749 (45)	$ 531 578 (49)	$ 7 532 168 (43)	$ 58 707 (44)
Total direct costs saving - N(%)	$ 22 750 411 (17)	$ 42 258 590 (14)	$ 24 899 359 (16)	$ 18 024 390 (14)	$ 79 434 704 (15)	$ 3 693 964 (13)
**Indirect costs (savings)**						
Pneumonia - N(%)	$ 3 522 333 (13)	$ 22 241 122 (11)	$ 1 400 094 (9)	$ 8 252 215 (14)	$ 8 557 686 (15)	$ 7 451 232 (15)
AOM - N(%)	$ 14 517 543 (20)	$ 9 740 639 (13)	$ 23 286 312 (17)	$ 11 218 127 (14)	$ 6 612 602 (15)	$ 10 446 078 (13)
Pneumococcal meningitis- N(%)	$ 774 859 (47)	$ 19 723 810 (48)	$ 905 497 (41)	$ 2 707 444 (50)	$ 925 147 (43)	$ 287 348 (35)
Pneumococcal bacteremia - N(%)	$ 703 551 (48)	$ 37 630 952 (48)	$ 299 735 (44)	$ 1 966 433 (51)	$ 10 054 366 (43)	$ 1 340 000 (47)
Total indirect costs - N(%)	$ 19 518 286 (19)	$ 89 336 523 (22)	$ 25 891 638 (17)	$ 24 144 218 (16)	$ 26 149 800 (21)	$ 19 524 658 (15)
**Cost of vaccination**						
Cost of vaccination	$ 58 299 995	$ 320 297 067	$ 21 768 674	$ 79 164 045	$ 168 367 142	$ 44 052 353

*Analysis is from birth till 10 years of age, discount rate 3.5. All costs are expressed in U$S Dollars. In Brazil, Colombia, Chile and México 2008 local costs were converted to US$ and in Argentina and Perú 2009 local costs were converted to US$ exchange rates are provided in methods.

% from total refers to the percentage of cases attributable to the condition from the total of cases or costs.

The largest effect in percentage reduction of cases was observed in pneumococcal meningitis (range 26.56% for Peru up to 47.44% for Colombia), neurologic sequelae after meningitis (range between 37.80% for Peru and 65.46% for Brazil) and bacteremia (range 42.34% for Argentina up to 49.34% for Colombia). Otherwise in absolute number of cases the largest effect was observed in pneumonia and AOM. Also, the model showed a significant proportion of pneumococcal related deaths were averted annually after PHiD-CV introduction (range between 17.65% for Peru and 33.43% for Brazil). Overall, the health benefits achieved with PHiD-CV vaccination resulted in a lower QALY loss (range from 14.73% lower for Peru up to 26.28% for Brazil). The differences between countries could be explained due to differences in specific etiology and serotype distribution estimations. There were no much differences on pneumonia effect because its etiology was not considered in the model but differences were shown in otitis, meningitis and bacteremia based on considering its specific etiology.

In addition to the health benefits, the introduction of PHiD-CV averted costs when compared with non-vaccination. Reduction in total direct costs varied between 13% for Peru and 17% for Argentina, representing a range of cost offsets between 3.69 million dollars (Peru) and 22.7 millions (Argentina). Economic benefits for PHiD-CV were in general greatest for AOM, this may be explained because AOM was the major cost driver in the base case scenario. Brazil was the only country were the major cost driver was pneumonia. These differences could be attributed to differences in incidence and events costs estimations between countries.

At a cost of 20 US$ for vaccine dose for all countries, compared to non-vaccination, cost-effectiveness analysis demonstrated significant health benefits in favor of 10-valent pneumococcal vaccination implementation, with ICER values between being cost saving in Chile ICER -230.13 US$ per QALY gained (where the vaccination strategy was less costly than the no vaccination one) to 7,088.64 US$ per QALY gained in Brazil (Table 9). The cost saving ICER in Chile could be explained due to a greater estimation of disease burden in comparison to other countries in pneumonia and AOM cases being the intervention relatively more cost effective. Chilean pneumonia hospitalizations were obtained from national health statistics and AOM cases from a local Chilean study, maybe these higher values can be explained due to a more developed statistics system or in the case of AOM due to a more detection of cases because of active surveillance in the context of the study. This greater pneumonia and AOM disease burden estimation associated with the impact shown in the sensitivity analysis of the pneumonia hospitalization reduction parameter could lead to a cost saving ICER estimation in Chile. The results of the cost-effectiveness analysis under different scenarios (considering herd protection and indirect costs) are also presented (Table 10). As shown in the table, the incorporation of indirect costs had a greater influence than that of herd immunity (in the assumed scenario). Table 9 contains main results expressed in International Dollars (I$) [68] in comparison with 3 GDP threshold.

**Table 9:** Cost effectiveness analysis (International Dollars)

	**Argentina**	**Brazil**	**Chile**	**Colombia**	**Mexico**	**Peru**
**3xGDP per capita threshold**	$ 44,116.90	$ 29,327.99	$ 42,261.21	$ 24,260.33	$ 42,347.63	$ 26,420.95
**ICER - QALYs gained**	$ 6,629.23	$ 8,354.35	$ -320.44	$ 6,518.50	$ 6,358.39	$ 5,986.93
**ICER - Life years gained**	$ 27,995.01	$ 9,496.72	$ -5,290.74	$ 13,513.13	$ 7,991.33	$ 12,881.14

3xGDPs are expressed in 2009 International Dollars with the following rates, for Argentina we used 1 I$ = 2 Argentine pesos, for Brazil we used 1 I$ = 1.6 Reais, for Chile we used 1 I$ = 376.1 Chilean pesos, for Colombia we used 1 I$ = 1,228 Colombian pesos, for Mexico we used 1 I$ = 8.1 Mexican pesos and for Peru we used 1 I$ = 1.5 Nuevos soles.

**Table 10:** Cost effectiveness analysis (US Dollars)

	**Argentina**	**Brazil**	**Chile**	**Colombia**	**Mexico**	**Peru**
**Main analysis**						
**ICER - QALYs gained**	$ 3,347.53	$ 7,088.64	$ -230.13	$ 4,021.37	$ 4,594.33	$ 2,975.23
**ICER - Life years gained**	$ 14,136.50	$ 8,057.94	$ -3,799.67	$ 8,336.48	$ 5,774.23	$ 6,401.33
**3xGDP per capita threshold**	$ 22,277	$ 24,884	$ 27,021	$ 14,966	$ 30,598	$ 13,129
**Secondary analysis**						
**ICER - QALYs gained**						
**Base case + indirect costs**	$ 1,509.59	$ 4,810.99	$ -2,133.38	$ 2,433.32	$ 3,243.41	$ 1,535.87
**Base case + herd immunity**	$ 3,295.13	$ 6,293.05	-235.11	$ 3,885.80	$ 4,144.01	$ 2,920.73
**Base case + indirect effect + herd immunity**	$ 1,454.51	$ 4,024.69	-2,141.37	$ 2,295.77	$ 2,790.71	$ 1,478.95
**ICER - Life years gained**						
**Base case + indirect costs**	$ 6,374.94	$ 5,468.84	$ -35,223.99	$ 5,044.38	$ 4,076.37	$ 3,304.48
**Base case + herd immunity**	$ 13,439.35	$ 7,092.43	-3,511.26	$ 7,928.28	$ 5,059.29	$ 6,186.27
**Base case + indirect effect + herd immunity**	$ 5,932.31	$ 4,535.93	-31,980.90	$ 4,684.11	$ 3,307.09	$ 3,132.50

*ICER*, Incremental cost-effectiveness ratio *QALYs*, Quality-adjusted life-years *HP*: Herd protection *IC*, Indirect costs.

All costs are expressed in US$ Dollars. In Brazil, Colombia, Chile and México 2008 local costs converted to US$ and in Argentina and Peru 2009 local costs were converted to US$ exchange rates are provided in methods.

Sensitivity analyses performed on the ICER of PHiD-CV vaccination compared with non-vaccination is shown in Table 11. The most influential variables are listed. In general, variables related to AOM and pneumonia had the greatest impact on ICER in all countries except Brazil, where IPD related variables affected ICER most (these can be explained due to the high estimation of bacteremia in Brazil in comparison with others countries). Nonetheless, changes were never large enough to surpass 3 times GDP cost-effectiveness threshold. Even considering 1 GDP, most countries estimations were below this threshold indicating that cost effectiveness estimations were robust.

**Table 11:** Sensitivity analysis

**Country**	**Parameter**	**ICER range**^**#**^		3xGDP $*
**Argentina**	Efficacy in preventing NTHI AOM (CI 95%)	$6,689.27	$3,753.22	$ 22,277
	AOM - Etiology (Sp -/+20%, NTHI -/+ 40%)	$5,959.08	$3,724.97	
	AOM - Etiology (Sp -/+ 20%)	$5,701.40	$3,886.36	
	AOM -Disutility for hearing loss (CI 95%)	$5,622.58	$3,982.36	
	Efficacy in preventing Sp AOM (CI 95%)	$5,501.06	$3,989.9	
**Brazil**	Bacteremia-Incidence (+/-50%)	$10,397.98	$6,021.45	$ 24,884
	Efficacy in preventing hospitalization for pneumonia (CI 95%)	$9,393.87	$6,311.59	
	Meningitis-Incidence (-/+50%)	$8,758.52	$6,667.62	
	Vaccine efficacy ramp-up (-/+ 20%, CI 95%)	$8,526.38	$6,920.00	
	Efficacy in preventing invasive disease (CI 95%)	$8,035.13	$7,261.63	
**Colombia**	Efficacy in preventing hospitalization for pneumonia (CI 95%)	$5,208.56	$3,195.50	$ 14,966
	Efficacy in preventing NTHI AOM (CI 95%)	$5,039.76	$3,502.43	
	AOM - Etiology (Sp -/+20%, NTHI -/+ 40%)	$4,864.28	$3,381.48	
	AOM - Etiology (Sp -/+ 20%)	$4,775.73	$3,433.84	
	AOM -Disutility for hearing loss (CI 95%)	$4,781.43	$3,469.80	
**Mexico**	Efficacy in preventing NTHI AOM (CI 95%)	$6,909.40	$3,408.91	$ 30,598
	Efficacy in preventing hospitalization for pneumonia (CI 95%)	$6,638.91	$3,181.98	
	AOM - Etiology (Sp -/+20%, NTHI -/+ 40%)	$5,830.11	$3,564.65	
	Bacteremia-Incidence (+/-50%)	$5,894.39	$3,704.39	
	AOM - Incidence (Myringotomies -/+ 20%, Total Cases -/+ 50%)	$5,364.50	$3,839.01	
**Peru**	Efficacy in preventing hospitalization for pneumonia (CI 95%)	$3,813.47	$2,422.55	$ 13,129
	AOM -Disutility for hearing loss (CI 95%)	$3,649.05	$2,511.47	
	Efficacy in preventing NTHI AOM (CI 95%)	$3,706.67	$2,617.21	
	AOM - Etiology (Sp -/+20%, NTHI -/+ 40%)	$3,577.81	$2,535.13	
	AOM - Etiology (Sp -/+ 20%)	$3,513.24	$2,570.60	

* 3GDP per capita threshold: 3 times Gross Domestic Product per capita. Values are expressed in U$S Dollars, conversion rates are provided in methods.

^#^ICER expressed per QALY gained.

## Conclusions

The initial economic evaluations of pneumococcal conjugated vaccines provided conflicting results. A review of studies in Europe and the US by De Graeve et al. [69] concluded that economic attractiveness of universal PCV vaccination strategies were dependent on both vaccine cost and willingness of decision makers to adopt a societal perspective. Similarly, Beutels et al. published a review of 15 economic analyses of heptavalent pneumococcal conjugate vaccine [70] and found that the cost of the vaccination program and the impact of herd immunity were highly influential parameters for cost effectiveness. Those studies considered high vaccine prices per dose and do not include AOM in the analysis. More recent studies in Latin America estimated PCV7 to be cost effective for the overall region [71] and for Brazil, Chile and Uruguay [56] considering AOM in their evaluated outcomes.

The results of the present analysis show that the introduction of the 10-valent pneumococcal non-typable *Haemophilus influenzae* protein D-conjugate vaccine into routine infant immunization programs in Latin American countries can significantly reduce the health and economic burden of pneumococcal disease. Vaccination with PHiD-CV in a 3 + 1 schedule at 20 US$ per dose proved to be a cost effective intervention, according to international criteria, in all countries analyzed. This result was achieved mainly due to the high disease burden averted (high pneumococcal serotypes coverage and protection against NTHi associated AOM) and the vaccine cost estimated in the base case scenario. In addition, the results obtained in this analysis proved to be robust, and the cost effectiveness of the vaccine was below the 3 GDP per capita threshold based on WHO standards [67], over a broad range of values for variables included in sensitivity analysis. In Brazil alone, the results were slightly above 1 GDP per capita while in the other countries the results were below this conservative threshold. The project lasted for many years and the most difficult challenge was to obtain model parameters prioritizing the use of local epidemiological data and costs.

Economic analyses in the Latin American region are often hindered by lack of epidemiological data. We conducted an extensive search of the literature to identify the best available data, validated by local experts for this study. Data reliability is crucial for accurate determination of outcomes. As an example, sources for AOM incidence rates were very scarce in our region and as mentioned, they have a great impact on study results. Surveillance studies usually overestimate AOM incidence rates while case report studies tend to underestimate them. A study of AOM in Boston children, reported an annual incidence rate of 120,000 per 100,000 children under 1 year of age [72]. Conversely, Arévalo et al. found an incidence rate of 1,260 per 100,000 children aged < 1 year and 1,083 per 100,000 children aged 1 to 4 years in spontaneously reported cases of AOM in Mexico [73]. Melegaro et al. have reported an annual AOM consultation rate of 24,000 per 100,000 children aged < 4 years [74] in the United Kingdom. Overall, we have used conservative AOM incidence rates based on three different local sources. In addition, some of the epidemiological data used were obtained from national health statistics and in absence of information, we based our analysis on expert opinion and Delphi panels. On the other hand, utility and disutility values were obtained from international sources due to lack of local data. Regarding model assumptions, as etiology of pneumonia cases is not fully understood, we used a vaccine effectiveness estimate for all-cause pneumonia from a US clinical trial of PCV-7 for all countries. As explained, the variations observed in the percentage of cases and deaths averted between countries are based on the different specific etiology and serotype distribution estimations assumed for each country. We conducted an extensive one way sensitivity analysis and were able to conclude that our results are quite robust, although a study limitation that may underestimate decision uncertainty is that a probabilistic sensitivity analysis was not included [75].

Some limitations of this study are that we did not include a comparison against other pneumococcal vaccines (at the time of the analysis the 13-valent vaccine was recently introduced in the market) and that we analyzed only one vaccination scheme (comparison information against different vaccination schemes was not available). Assuming that a 2 + 1 scheme would be close in efficacy to a 3 + 1 but at a lower cost, the results of our analysis are conservative. In addition this was an ambitious project that lasted many years and we are now presenting the first results in this manuscript. Publications with further analysis on different scenarios (schedules) per country and comparison between vaccines are in progress. A few comparative studies [76,77] on the cost effectiveness of pneumococcal conjugated vaccines were published recently, and their results are encouraging.

Although the current PAHO revolving fund vaccine price per dose is around 14 USD, we used 20 USD per dose in our analysis to assume a conservative scenario. In our study, the vaccine shows to be highly cost effective even in this scenario. In Latin America, once a vaccine is included in the PAHO revolving fund there is no price negotiation.

In this study we are presenting a cost effectiveness evaluation of the incorporation of the 10-valent pneumococcal conjugate vaccine for several Latin American countries. In a context where decisions are not always driven by evidence, our study provides a detailed description of many aspects of the disease burden estimation needed to carry out an economic evaluation, and shows that the adoption of the vaccine has the potential to become a valid strategy to reduce disease burden and infant mortality while optimizing the use of available and scarce resources in these developing countries.

## Competing interests

Authors from IECS had no conflict of interest or competing interest. JG and GK work at GSK.

## Authors’ contributions

GMS: Responsible for the coordination of IECS teams on data collection, data analysis, modeling work and manuscript writing. Also participate actively in the discussions with local country investigators for final model calibration. CL: Responsible of modeling work for each country and participate in data analysis and manuscript writing. BA: Responsible of epidemiological data collection for each country and participate in data analysis. GJ: Participated actively in modeling work and data analysis for each country. LA: Responsible of development, administration and data analysis from Delphi panels for each country, to gather data not available from other sources. CJ: Responsible of cost data collection and estimation for each country. AF: Overview of the project at IECS with active participation in modeling work, data analysis and manuscript preparation. P-RA: Overview of the project at IECS with active participation in modeling work, data analysis and manuscript preparation. GK: Responsible of model design, and model development at GSK with active participation on data analysis and manuscript preparation. GJ: Overview of the whole project with active participation on data collection, discussion with local country investigators for model calibration, data analysis and manuscript preparation. All authors read and approved the final manuscript.
